# Bezold Abscess: A Case Report

**DOI:** 10.5811/cpcem.62179

**Published:** 2026-09-08

**Authors:** John Wahhab, Ani Oganesyan, Peter Pham, Evan L. Jackson

**Affiliations:** * Chicago Medical School at Rosalind Franklin University of Medicine and Science, Department of Medicine, North Chicago, Illinois; † University of Illinois Chicago, Department of Emergency Medicine, Chicago, Illinois

**Keywords:** Bezold abscess, mastoiditis, neck abscess, computed tomography, case report

## Abstract

**Introduction:**

A Bezold abscess is a neck abscess characterized by inflammation and infection of the mastoid process that can invade deep into local anatomical musculature and vasculature.

**Case Report:**

We highlight the case of a 62-year-old man who presented with ongoing left-sided neck pain, headache, and tinnitus that started two weeks prior to arriving at the emergency department. Physical examination showed tenderness to the left trapezius muscle, left-sided mastoid tenderness, and pearly tympanic membranes bilaterally. A computed tomography angiogram of the head and neck was performed due to concerns of vertebral dissection, which eventually led to a diagnosis of Bezold abscess.

**Conclusion:**

Although Bezold abscess has been described in the medical literature, it is rarely encountered in modern clinical practice because the widespread use of antibiotic therapy prevents acute mastoiditis from progressing to abscess formation. We highlight the complexity of clinical presentation that can easily be mistaken for other medical conditions, such as occurred with our patient who presented with symptoms more consistent with vertebral dissection: neck pain; tinnitus; and headache, without associated ear pain or discharge.

## INTRODUCTION

Bezold abscess is characterized by infection and inflammation of the mastoid process tip that often produces a suppurative collection of pus in the digastric muscle.^1^ If Bezold abscess is left untreated, purulent material will accumulate in the mastoid bone causing osteitis and osteonecrosis of fragilized pneumatized air cells.^2^ Pneumatized air cells are cavities of air-filled spaces through the eustachian tube and middle ear that are responsible for offsetting pressure changes in the middle ear and permitting gas exchange within the submucosal capillary network.^3^ Purulent secretion from severe mastoiditis can extend into deeper and surrounding anatomical structures that may cause significant morbidity. However, the use of antibiotics therapy has significantly altered the clinical course of mastoiditis, rendering Bezold abscess a rare complication in modern practice.^1^

Risk factors for Bezold abscess include cholesteatoma, recurrent otitis media, or recurrent mastoiditis, which are conditions more common in the pediatric population.^1^ Normal growth into adulthood elongates the skull base and the eustachian tube becomes more oblique and better drained, decreasing infection risk.^4^ Additionally, pneumatized air cells decline with age and rapidly decrease during the seventh decade, reducing pathways for infection to spread into the mastoid.^5^ Consequently, the likelihood of Bezold abscess formation in elderly patients is significantly reduced, which can cause underrecognition of Bezold abscess as a differential diagnosis and consequently create delays in treatment.

Since Bezold abscess is not commonly encountered in clinical practice, we chose to inform physicians about this rare diagnosis and its treatment to prevent morbidity and mortality. This case report highlights the importance of considering Bezold abscess in the differential diagnosis of neck pain, even in patients who fall outside the typical pediatric age range and lack traditional risk factors.

## CASE REPORT

A 62-year-old man presented to the emergency department with two weeks of left-sided neck pain, headache, and tinnitus that began while shoveling snow. His past medical history included previous subarachnoid hemorrhage and alcohol use disorder. His initial vital signs showed the following: blood pressure, 128/84 mm Hg; heart rate, 103 beats/min; oxygen saturation, 95% on room air; respiratory rate, 18 breaths/min; and temperature, 36.1°C. His physical examination showed mild tenderness to palpation in the left trapezius muscle and the midline of the neck, mild left-sided mastoid tenderness, and pearly tympanic membranes bilaterally. Cerebellar function was intact to finger-to-nose and rapid alternating movements.

The patient’s left-sided neck pain, tenderness, and tinnitus prompted concern for vertebral artery dissection. Differentials such as ischemic stroke, intracranial hemorrhage, infection, and other neurological catastrophe were also considered due to his mastoid tenderness and previous history of subarachnoid hemorrhage. Therefore, computed tomography (CT) angiogram of the head and neck was performed, which demonstrated asymmetric soft tissue fullness with central hypoattenuation in the high cervical soft tissues underlying the left mastoid, appearing to extend into the left lateral epidural space (Images 1–3). These findings were concerning for otomastoiditis with associated Bezold abscess given concomitant osseous erosion. Otolaryngology was consulted and recommended intravenous antibiotic therapy and transfer to a tertiary care center for a higher level of care. The patient received vancomycin (1,750 mg), ampicillin-sulbactam (3g) intravenously and was transferred to an academic center.

At the university hospital, the patient was evaluated by the otolaryngology service, which recommended further imaging with magnetic resonance imaging and consultation of infectious diseases and interventional radiology. The patient was admitted to the surgical intensive care unit and started on vancomycin (1,500 mg) every 8 hours and cefepime (2 g) IV every 8 hours.

*CPC-EM Capsule*

What do we already know about this clinical entity?

*Bezold abscess is a rare mastoiditis complication causing deep neck infection, classically seen in pediatric patients with otologic symptoms.*

What makes this presentation of disease reportable?

*An elderly patient presented with neck pain and tinnitus concerning for vertebral artery dissection but was ultimately diagnosed with Bezold abscess.*

What is the major learning point?

*Bezold abscess can present atypically in older adults; persistent neck pain with mastoid tenderness may warrant advanced imaging.*

How might this improve emergency medicine practice?

*Recognizing atypical Bezold abscess presentations may reduce diagnostic delays and prevent severe infectious complications.*

Magnetic resonance imaging revealed malignant otitis externa, osteomyelitis of the skull base, a 16-mm abscess, and a dural venous thrombosis. Interventional radiology was consulted but determined they were unable to drain the abscess. Otolaryngology performed a left myringotomy with tympanocentesis, and cultures were sent. Culture results yielded the following: *Rothia mucilaginosa; Actinomyces; Streptococcus mitosis; Streptococcus oralis;* and *Prevotella*. The infectious diseases service recommended a six-week duration of antibiotics. Due to the dural venous thrombosis, the patient was started on a heparin drip and eventually bridged to warfarin.

After a prolonged hospital course, a repeat CT performed on an inpatient basis showed resolution of the fluid collection. The patient was then discharged home to continue a course of amoxicillin-clavulanate (875–125 mg) twice per day. Otolaryngology recommended audiology testing, and results were noted to be unremarkable. The patient followed up with otolaryngology and infectious diseases in the outpatient clinic and noted full resolution of his symptoms.

## DISCUSSION

The novelty of this case lies in its presentation of an elderly patient without traditional risk factors and with nonotologic symptoms that initially suggested a vascular cause. Bezold abscess is an exceptionally rare infectious complication of mastoiditis, with fewer than 100 reported cases in the literature.^6^ This condition typically presents in children and young adults with tenderness below the mastoid process, neck swelling, restricted neck movement, ear pain and discharge, and fever.^7^ In contrast, this patient’s symptoms were initially more suggestive of vertebral artery dissection, underscoring the complexity of Bezold abscess in older adults. The patient’s physical examination findings of low temperature (36.1 °C) and pearly tympanic membranes did not match the classic Bezold abscess findings of fever and tympanic membrane changes associated with infection.^8^ Furthermore, he lacked common risk factors such as a history of cholesteatoma, chronic otitis media, or recurrent mastoiditis.^1^

Advanced imaging with CT angiography was pursued over more conservative initial modalities due to the patient’s persistent neck pain over several weeks and associated neurologic findings, including tinnitus, which raised concern for vertebral artery injury. This case highlights the importance of escalating evaluation when symptoms are persistent, progressive, or accompanied by atypical otologic or neurologic features despite an otherwise nonspecific presentation. Physicians should maintain a low threshold for advanced imaging in patients with a history of cholesteatoma, recurrent otitis media, or mastoiditis who present with persistent neck pain in conjunction with mastoid tenderness, hearing changes, neck swelling, or neurologic symptoms, because these findings may indicate serious pathologies such as Bezold abscess.

Although Bezold abscess is traditionally considered rare in older adults due to age-related loss of pneumatized mastoid air cells, emerging reports suggest it may be underrecognized in this population. The condition has been reported in a growing number of elderly male patients, reflecting a need to include it in the differential diagnosis alongside other entities such as deep neck abscesses, suppurative parotitis, coalescent mastoiditis, malignancy, and cutaneous abscesses.^9^

## CONCLUSION

The severity and complexity of this patient’s findings required a multidisciplinary approach involving specialists from otolaryngology, infectious diseases, interventional radiology, and hematology. By increasing clinical awareness and prioritizing early collaborative management, physicians can significantly improve patient outcomes and reduce the risk of severe complications associated with Bezold abscess.

## Figures and Tables

**Image 1:**
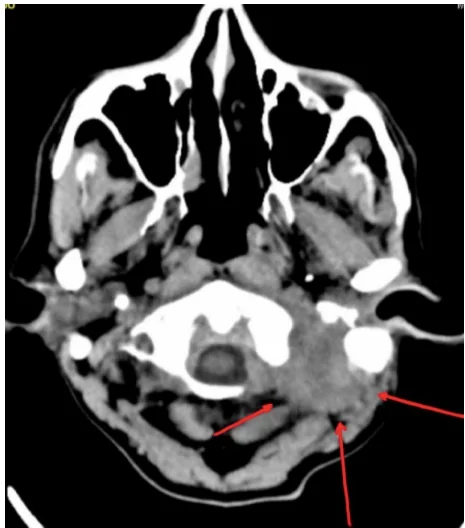
Axial computed tomography angiogram of the head and neck demonstrating a 3.0 x 3.7 x 3.1 cm ill-defined soft tissue density (arrows) centered inferior to the medial aspect of the mastoid temporal bone and lateral to the left occipital condyle with disruption of the fat planes and a central hypodensity concerning for Bezold abscess.

**Image 2:**
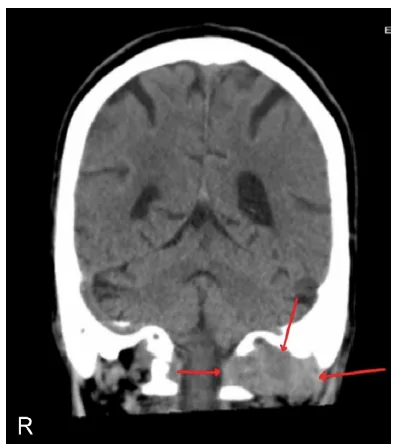
Coronal computed tomography angiogram of the head and neck demonstrating a 3.0 x 3.7 x 3.1 cm soft tissue density (arrows) located inferior and medial to the left mastoid process of the temporal bone and extending to the occipital condyle. There is an additional central hypodense region, which is a collection consistent with fluid or pus, concerning for Bezold abscess.

**Image 3:**
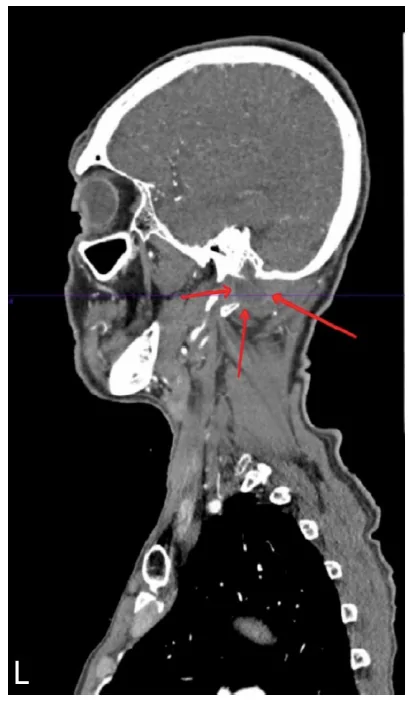
Sagittal computed tomography angiogram of the head and neck demonstrating a 3.0 x 3.7 x 3.1 cm (arrows) abnormal soft tissue collection located inferior and posterior to the mastoid process with extension toward the cervical musculature. Within the central region is a hypodense cavity suggestive of fluid or abscess formation, accompanied by adjacent inflammatory changes to fascial planes, concerning for Bezold abscess.
